# Workshop Report: Catalyzing Knowledge-Driven Discovery in Environmental Health Sciences through a Harmonized Language

**DOI:** 10.3390/ijerph20032317

**Published:** 2023-01-28

**Authors:** Stephanie Holmgren, Shannon M. Bell, Jessica Wignall, Christopher G. Duncan, Richard K. Kwok, Ryan Cronk, Kimberly Osborn, Steven Black, Anne Thessen, Charles Schmitt

**Affiliations:** 1 Office of Data Science, National Institute of Environmental Health Sciences (NIEHS), Durham, NC 27709, USA; 2 Research Triangle Institute, Durham, NC 27709, USA; 3 Health Sciences, ICF, Reston, VA 20190, USA; 4 Genes, Environment, and Health Branch, Division of Extramural Research and Training, National Institute of Environmental Health Sciences (NIEHS), Durham, NC 27709, USA; 5 Division of Neuroscience, National Institute on Aging (NIA), Bethesda, MD 20892, USA; 6 Center for Health Artificial Intelligence, University of Colorado Anschutz Medical Campus, Aurora, CO 80045, USA

**Keywords:** FAIR data, harmonized language, impact network, ontology

## Abstract

Harmonized language is essential to finding, sharing, and reusing large-scale, complex data. Gaps and barriers prevent the adoption of harmonized language approaches in environmental health sciences (EHS). To address this, the National Institute of Environmental Health Sciences and partners created the Environmental Health Language Collaborative (EHLC). The purpose of EHLC is to facilitate a community-driven effort to advance the development and adoption of harmonized language approaches in EHS. EHLC is a forum to pinpoint language harmonization gaps, to facilitate the development of, raise awareness of, and encourage the use of harmonization approaches and tools, and to develop new standards and recommendations. To ensure that EHLC’s focus and structure would be sustainable long-term and meet the needs of the field, EHLC launched an inaugural workshop in September 2021 focused on “Developing Sustainable Language Solutions” and “Building a Sustainable Community”. When the attendees were surveyed, 91% said harmonized language solutions would be of high value/benefit, and 60% agreed to continue contributing to EHLC efforts. Based on workshop discussions, future activities will focus on targeted collaborative use-case working groups in addition to offering education and training on ontologies, metadata, and standards, and developing an EHS language resource portal.

## 1. Introduction

Environmental health sciences (EHS) data are being generated at a rapid rate. Leveraging these data with a harmonized language is essential to answer large-scale complex questions and to increase data finding, sharing, and reuse. A harmonized language combines multiple languages into a single comparable view, building from the components of each language. For example, if two pathologists use different sets of terms to describe lesions, one could compare findings across the pathologists if the terms from each can be mapped to a single harmonized set of terms. The benefits of a harmonized language to describe research data, express scientific concepts and findings, and communicate within and between scientific fields are well-established [1]. In response, multiple efforts have worked towards defining language standards and recommendations across subfields of science and medicine [2,3,4,5,6]. Related efforts have focused on the development of tools to facilitate the use of a standard language [7,8,9,10,11,12,13,14]. Despite these efforts, considerable gaps exist in the adoption of harmonized language approaches within the field of environmental health [15,16,17].

These gaps exist for a variety of reasons. In part, the breadth and diversity of the field hampers the adoption of a common language across subfields. “Environmental health” covers physical, biological, chemical, social, spatial, and psychosocial factors that may impact human health and span a variety of subfields, such as exposure science, epidemiology, biological sciences, toxicology, medical sciences, statistics, geography, psychology, and sociology. The community of people involved in environmental health is equally diverse, covering physical and life scientists, computer and information scientists, regulators, policy analysts, health practitioners, advocacy groups, industry, and the public. The gap in the adoption of harmonized language is exacerbated by the ongoing evolution of environmental health practices, driven by emerging areas of concern, such as climate change; advances in technologies, such as machine learning or smart device capabilities; or improvements in methodologies, such as the adoption of new survey instruments. Developing and adapting harmonized language to these changes rarely matches the pace of scientific practice.

**Figure 1:**
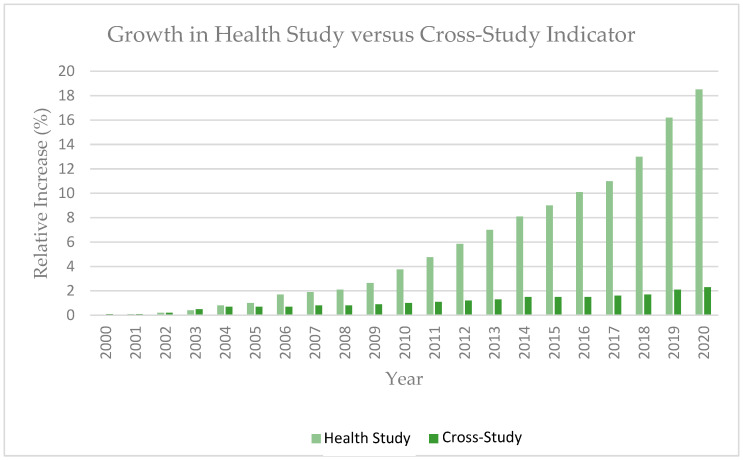
Relative increase in cross-study analyses compared to the relative increase in health study publications indexed in PubMed since 2000 [18]. Cross-study analyses were identified by searching for “meta-analysis”, “meta-analyses”, or “systematic review”.

While the challenges are not trivial, the benefits that arise from the use of harmonized language are critical for advancing scientific discovery. Scientific inquiry depends on the comparison of findings and triangulation of evidence to support new discoveries and decisions. Without harmonized language, efforts to look across studies through pooling, meta-analysis, and systematic reviews often require extensive time and resources from researchers to compare and map measures, outcomes, and findings into comparable concepts. Figure 1 shows that, although PubMed-indexed health study publications have increased significantly over time, the relative increase in cross-study analyses is much lower. Additionally, the inability to create definitive mappings reduces the scientific value of the efforts.

The benefits of harmonized language extend beyond support for cross-study analysis. The interpretation of scientific data and the ability to generate hypotheses are increasingly enabled by knowledge organization systems [19,20] that bring together EHS knowledge into computational frameworks. These can include computer representations of signaling or adverse outcome pathways (AOPs), ontologies such as the Gene Ontology, and integrated knowledge bases such as the Comparative Toxicogenomics Database (CTD) 6 or the Monarch Initiative [21]. Knowledge organization systems are increasingly important for research strategies as the quantity of EHS knowledge and data is too large for comprehensive human review. Advanced knowledge systems provide methods to mine and generate inferences over extremely large sets of data [22,23,24,25]. These systems depend on the use of standardized terminologies and ontologies to represent knowledge and harmonized language, to map data to knowledge representations.

Recent activities in EHS demonstrate how harmonized language can increase the research impact and effectiveness. For example, exposome projects seek to measure and assess the totality of internal and external exposures during the lifetime of an individual [26] and relate those exposures to health outcomes. To enable this research, new composite exposomic datasets will be needed that integrate omics measures with measures that include exposures and outcomes often collected from different studies and researchers. Another example is incorporating geospatial-related data into analyses that consider disparities in health impacts due to climate change-related exposures. This research will require the use of a harmonized language to integrate measures generated from multiple study cohorts and mechanistic studies. Finally, advances in data science and analytics allow for powerful predictive capabilities. Developments in fields such as computational linguistics and computer vision enable machine algorithms to extract information from complex sources such as literature and images.

To foster the development and adoption of a harmonized language for EHS, the National Institute of Environmental Health Sciences (NIEHS) and partners have created the Environmental Health Language Collaborative (EHLC, or the Collaborative). The genesis behind the Collaborative was a recognition among multiple individuals and groups of the need for harmonized language and the acknowledgment that past progress had been hampered by the lack of a sustained community effort to identify, prioritize, and address gaps.

This Collaborative builds on work begun at an NIEHS-sponsored event, “Workshop for the Development of a Framework for Environmental Health Science Language” (workshop proceedings) [16], held in September 2014, and work achieved at the 2019 Computable Exposures Workshop [17]. While these workshops had productive outcomes, discussion from both events determined that occasional gatherings were insufficient to address the challenge and that the EHS field could benefit from a sustained, community-driven effort. As a result, EHLC was conceived to provide the administrative and strategic infrastructure to enable experts from a variety of fields and organizations to collaborate on developing and promoting the adoption of harmonized language approaches within environmental health and toxicology.

The intent of EHLC is to establish a forum for the environmental health community to:

- Create a central space to engage the diversity of expertise in the environmental health community around harmonized language.
- Raise awareness of efforts that exist related to harmonizing language to reduce redundancy and promote the adoption of existing tools and approaches.
- Identify opportunities within those efforts to extend them to meet new needs or uses.
- Seek synergies across existing efforts to maximize their benefit.
- Promote the EHS community’s involvement in developing new standards/recommendations, especially in areas that are EHS-adjacent (e.g., earth sciences, ecology, clinical, behavioral sciences).
- Pinpoint gaps that need solutions and facilitate the development of solutions to address those gaps.

This article outlines activities from the September 2021 workshop related to establishing the community model for and outputs of the Collaborative, as well as planned upcoming activities for the Collaborative. It also outlines why the Collaborative is necessary and how an established community can benefit this research area more than individual meetings or workshops.

## 2. Methods: Workshop Overview

To begin the process of the necessary community building described previously, the Collaborative was launched in September 2021 at a two-day interactive virtual workshop, “Catalyzing Knowledge-driven Discovery in Environmental Health Sciences Through a Harmonized Language” (Workshop Recordings) [27]. In the year leading up to the workshop, a series of planning meetings and community engagement activities were conducted to prepare for and design the workshop around two themes:

1. **Developing Sustainable Language Solutions:** Identify use cases in EHS research and begin specifying semantic needs, gaps, and strategies for developing and implementing solutions.
2. **Building a Sustainable Community:** As the Collaborative is intended to be a community-driven initiative, three workshop sessions were dedicated to start the process of attaining agreement on the proposed EHLC purpose, community model, and strategy to build a sustainable and impactful community.

To promote productive workshop discussions, attendees were encouraged to prepare before the workshop and asked to:

- Review the workshop booklet [28]. Attendees were asked to reflect on the Questions to Ponder as well as become familiar with semantic terms and concepts.
- Read through the use-case profiles and prepare to participate in the use-case “work-a-thon” sessions.
- View pre-workshop webinars, The Value of Creating Language and Community in Catalyzing Knowledge-Driven Discovery in Environmental Health Research (June) [29] and A Primer on Using Terminologies, Vocabularies, and Ontologies for Knowledge Organization (July) [30]. June’s webinar addressed the value of language and community and raised awareness of the Collaborative, to begin collecting community input. July’s webinar explained the differences between a taxonomy, a thesaurus, and an ontology, where to find ontologies, and when and how to use them.

**Figure 2:**
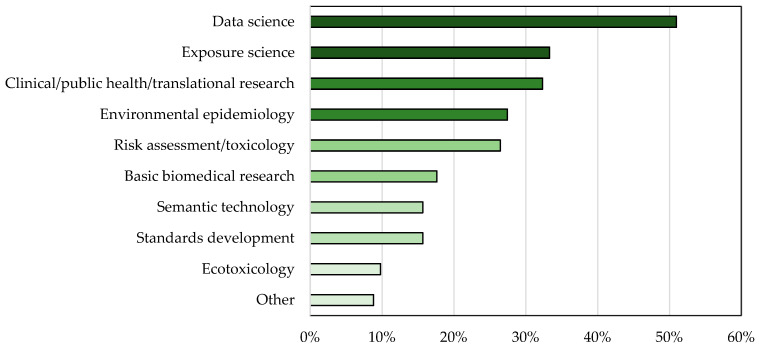
Research areas and disciplines of September 2021 EHLC workshop participants.

**Figure 3:**
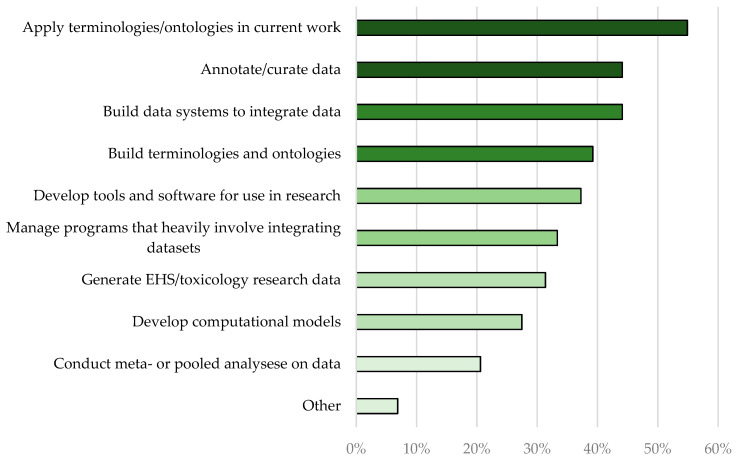
Proportions of workshop participants interested in the proposed activities.

The workshop was held on September 9 and 10, 2021, virtually using Zoom and MURAL. MURAL is a digital whiteboard useful for virtual collaboration and brainstorming. The total number of participants was 204, with more than 100 attending both days. Attendees who provided contact details represented governmental institutions (approximately 40% of participants), academia (approximately 33%), and other groups including nonprofits and industry (approximately 17%). The participants reflected a broad range of disciplines (Figure 2) and had a variety of reasons for participating in the workshop (Figure 3).

**Figure 4:**
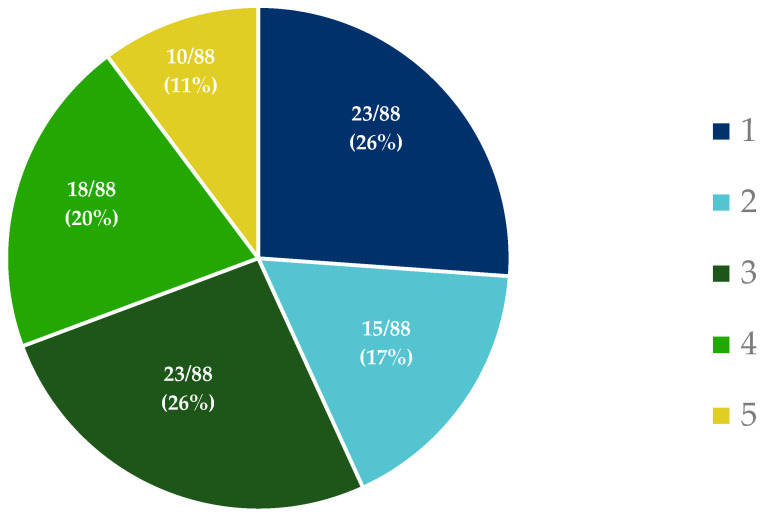
Participants’ understanding of terminology. Responses to the workshop poll question on a scale from 1 (low) to 5 (high), “How well do you understand the differences between controlled vocabulary, thesaurus, and ontology and when to use one versus the other?”

In addition, attendees were polled on their familiarity with the differences between a terminology, thesaurus, and ontology (Figure 4). On a scale from 1 (low) to 5 (high), 30% rated their understanding at 4 or 5, and 43% rated their understanding at 1 or 2, with the remaining 26% in the middle at 3.

To begin the discussion around developing semantic solutions, brief overviews of the five use cases developed for the workshop were presented to help attendees determine which work-a-thon sessions they would like to attend. At the work-a-thon sessions, participants further defined the use cases and developed action plans for the next steps. A summary of each use-case discussion was provided at the report-out session. In addition, participants had the opportunity to attend a community feedback session using MURAL to identify semantic needs, gaps, and strategies that EHLC could consider.

For the community building theme, the proposed Collaborative vision, mission, goals, and community model were presented to facilitate discussion and begin the process of achieving community endorsement for EHLC’s purpose and structure. A separate MURAL session focused on obtaining answers to questions related to how participants want to define success for the Collaborative and potential barriers to success.

## 3. Theme 1: Developing Sustainable Language Solutions

### 3.1. Goals of the Theme

The goals of the use-case workshop sessions were to:

- Identify use cases in which the community felt the development or adoption of a harmonized language, including terminologies, ontologies, common data elements, and supporting tools, was needed.
- Gather information on use cases of interest to the community and the willingness of community members to participate in use-case working groups (WGs).
- Make progress on use cases identified in the prior year and develop action plans for continuing the work post-workshop.

### 3.2. Value of Use Cases

Use cases were developed to serve as the foundation for the work to be carried out within the Collaborative as they help scope out what is necessary to address real-world needs. A use case is defined as a “narrative written from the perspective of a user trying to complete a task in the proposed infrastructure. They are used to help constrain the features of the system so that the development effort is focused on a user need” [17].

Use cases can spark the development of new research capabilities to address needs and data gaps. They can provide support for funding initiatives, can be used as training materials for workforce development, or can help those building data systems to stress test whether the system is properly designed to answer the question or problem posed by the use case.

Use cases are intended to provide a means of communicating between different scientific communities, to enable practices and technologies and allow for collective examination of:

- Terminology and ontology gaps that impede research goals.
- Challenges in advancing harmonized languages.
- Opportunities for advancing the creation and adoption of terminologies and ontologies.

### 3.3. Development of Use Cases

In early 2020, a small WG drafted several use cases and sub-use cases as starting points for potential activities of the Collaborative. Subject matter experts from the EHS community volunteered to “champion” one of five use cases and lead a WG to make progress on addressing the use case.

During the summer, each of the use-case WGs met to draft a “use-case package” to be put forward as the basis of discussion at the September workshop. The use-case package included the proposed goals and scope of the use case, suggested resources (datasets, standards, tools) that could support the use case, identified gaps and challenges, and recommended reading to provide background.

The discussions in pre-workshop use-case meetings led to the decision to merge two of the use cases, resulting in four use cases that moved forward.

At the workshop, each of the champions led 1.5 h work-a-thon sessions during which the initial use-case package was presented, and participants engaged in discussion to further refine the use case and begin outlining action plans and next steps. Further details on each use case are provided below (Table 1).

### 3.4. Use Case: Discovery of Exposure Data

#### 3.4.1. Background

Understanding the health impacts of environmental chemical exposures requires the ability to find and use information from multiple fields and integrate it to gain insights and avoid confusion or misleading conclusions. Finding data can be challenging as data are often in discipline-specific reports, and publications use different terminologies and formatting. Even when data are available in a computationally accessible format such as in databases, the additional context needed to assess the data’s relevance may not be available.

This case study focused on finding chemical toxicity data for a given exposure scenario. This included identifying metadata (such as units and how the data were generated) with sufficient context and ensuring that the terminology was clear outside of discipline-specific jargon. The necessary data discovery steps were defined as:

- Searching for existing data and identify gaps.
- Screening data for relevance and curate to add context.
- Integrating information.

#### 3.4.2. Workshop Discussion

Much of the work within environmental sciences, particularly toxicology, is carried out through small studies. With the goal of making this smaller-study data more accessible to the larger community, four key actors were identified whose diverse needs/roles related to the three data discovery steps. These are outlined in Table 2.

With these four actors in mind, the discussion focused on what each was trying to achieve and how each actor could aid in achieving the objectives. Key discussion points focused on incentivizing data annotation and making it easier for data generators to annotate data at the point of submission, encouraging the addition of sufficient detail to assess data’s relevance in searchable fields, and expanding technical tools to aid in finding and extracting data as well as automating the annotation of key terms.

#### 3.4.3. Results: Future Directions for This Use Case

Making environmental health data findable and accessible for the larger community is going to take effort from all actors. The use-case group identified some low-hanging fruit that could be a focus for more immediate improvements in finding data. This included the creation of a minimal information template to communicate what information should be provided in datasets or in article metadata when publishing a dataset and working with journals to adapt and enforce data accessibility policies. Longer-term efforts to provide structural support included expanding training and creating repositories and tools to support data structuring, annotation, and storage.

### 3.5. Use Case: Place-Based Exposures

#### 3.5.1. Background

Place-based research has been used extensively in EHS to estimate exposures and is a major component of climate change research. Using data sources, such as census information or historical records, beyond those examining chemical or physical exposures presents opportunities to integrate structural and societal factors that underlie exposures and characterize environmental injustice. Opportunities to integrate data from multiple place-based exposures and data from different studies across varied geographic locations are important to further understand how place influences health. The benefits of having a unified language for place-based environmental health research are numerous and include improved rigor, reproducibility, interpretation, and utility among the research community; data harmonization across studies; increased usability and adoption of existing datasets; increased diversity and geographic variation across studies to improve estimates and generalizability; and improved communication within and beyond the environmental health community. The goal is to develop tools and strategies for shared vocabulary and semantic ontologies that could improve the rigor and interoperability of place-based research, increasing the impact of the research to improve public health and inform prevention and policy efforts.

#### 3.5.2. Workshop Discussion

The discussion centered around the identification of common data elements that would allow for the linkage of disparate place-based research studies by using real-world published studies as a starting point. Based on group discussions, the focus of the use case was refined to consider the data involved in studying air pollution and asthma and further refined to focus on the exposure assessment portion of such a study rather than include the health outcomes, given parallel efforts on data harmonization in outcomes research.

Participants acknowledged the difficulty of defining the term “place-based” since many different contexts have been used. In addition, place-based data can be both static and dynamic and can be considered at various scales and levels of aggregation. For instance, air pollution measures are based on a fixed sensor over a time series. Others, such as census information on neighborhood characteristics, are point or polygon data and are relatively static. Still others can be detailed to the individual level. Location can also have both quantitative and qualitative meanings, for instance, a hierarchy of global > country > subregion > admin boundary > home > room > geocoordinate versus a qualitative meaning that associates geography or location with a function (e.g., home, school, daycare). In the end, it is important to identify a minimum set of variables and to consider both space and time together, to harmonize data across studies and reduce the burden on the data provider.

Several barriers and challenges were identified. Administrative units vary greatly, and how data are aggregated and divided is not standardized. Adopting a standard for encoding [31] rules would be helpful. A huge barrier is the linking of exposure data and health data, which often involve regulatory and privacy barriers to access. It is important to assess existing standards given the implications of harmonization and avoid over-categorization of ontologies, to allow for greater integration and move toward interoperability.

#### 3.5.3. Results: Future Directions for This Use Case

Participants discussed connecting with existing ontologies such as Open Biological and Biomedical Ontology (OBO) Foundry ontologies, Observational Health Data Sciences and Informatics (OHDSI), and Canadian Urban Environmental Health Research Consortium (CANUE) [32,33,34,35,36,37,38,39,40] and performing a landscape analysis to identify existing ontologies and how best to incorporate them into the environmental health field. These examples need to be use-case-driven (in both exposure science and social determinants of health) so that we can learn from the studies, test out the vocabulary and better determine how they can be generalized to the field.

### 3.6. Use Case: Integration of Exposure Data

#### 3.6.1. Background

The overall purpose of this use case is to enable the integration of individual-level data from multiple independent studies, to inform how one or more environmental exposures affect one or more health outcomes. To achieve this goal, the use-case team will pursue the development of solutions that leverage semantic tools such as ontologies and knowledge graphs in the collection and analysis of data, to make it easier to combine individual-level data for pooled analyses. This use case is framed from the perspective of a researcher (or research team) conducting analyses, to uncover an etiologic relationship between an exposure (or combination of exposures) and a health outcome. During the workshop, use-case participants further refined the scope to focus primarily on future/prospective data collection rather than retrospective data harmonization. The group agreed to develop one or two demonstration use cases around prospective data collection. Through these examples, the goal is to highlight different potential metadata needs and create modular templates to support layers at different levels of discovery. Importantly, these templates should be annotated with existing ontologies and should leverage and build on the existing work and requirements of large programs, such as the Human Health Exposure Analysis Resource (HHEAR) [41] and Environmental Influences on Child Health Outcomes (ECHO) [42]. Through this use case, the team expects to illustrate what research studies gain from structured reporting of metadata and what they lose by not reporting metadata.

#### 3.6.2. Workshop Discussion

Discussion at the workshop centered around identifying gaps in metadata reporting and knowledge representation that hinder the harmonization of data across studies and prevent the use of semantic and other technologies. Several key points were raised in the group discussions. First and foremost, data must be accessible. For a cross-study harmonization effort, it is of utmost importance to have the right information in an accessible location/repository. Even if one has to restructure data extraction on-the-fly, an accessible, complete dataset is preferable to an incomplete or inaccessible dataset with an ideal structure. Second, the group stressed the importance of conveying the experimental context for the dataset through descriptive metadata. To fully use datasets for pooled analysis, extensive metadata are needed that describe the context of what is being measured (e.g., experimental details). Ultimately, the group encouraged every publication and dataset to have some standard metadata. While meeting these standards is challenging, the group stressed the importance of not “reinventing the wheel”. There are many parallels between a meta-analysis and systematic review, including search strategies, inclusion/exclusion criteria, and quality scoring, and borrowing relevant approaches for reporting is necessary.

Throughout the use-case discussion, the group noted several gaps that need to be addressed to achieve the use-case goals. These gaps included the need to develop:

- Methods and best practices for reporting machine-readable data and metadata.
- Standards for reporting, including templates to report information in a common format.
- Standardized structures for reporting metadata that include requiring metadata to be reported at both the study level and the variable level.

The group highlighted several challenges in addressing these gaps. First, there are so many potential users of a given dataset that it is challenging to encompass all potential users’ needs. The group suggested compiling a list of key uses/user personas. Secondly, in our current paradigm, metadata files often do not include all the details that are needed for harmonization. Metadata associated with lab data, such as analytical instrument details (e.g., run dates), are difficult to acquire for many studies, especially those that are small or employ novel assays. Additionally, there is an ongoing challenge with sparse and incomplete datasets.

#### 3.6.3. Results: Future Directions for This Use Case

The final desired output of this use case is the development of tools and strategies to facilitate data sharing and harmonization through the use of data and metadata standards and the annotation of existing datasets. To achieve the desired output, the group outlined several “next steps”.

Moving forward, the use-case team plans to refine the use-case definition with a focus on future/prospective data collection rather than retrospective data harmonization. Future plans include conducting outreach to relevant stakeholders, such as Clinical and Translational Science Awards (CTSA) [43] groups and journals (e.g., Environmental Health Perspectives [44]), to improve the data harmonization strategy’s uptake and implementation.

Initial work plans include:

- Developing initial lists/templates of standard structured metadata for various environmental health studies. The guiding principles include being modular, extensible, and lightweight; having different templates for different studies; and including required variables and desired variables. Importantly, elements should be linked to ontological terms.
- Performing landscape/mapping analysis of desired variables to existing ontologies.

### 3.7. Use Case: Bridging Exposure and Biomarkers of Exposure

#### 3.7.1. Background

This use case is intended to explore semantics for capturing the biomarkers and biological processes perturbed by an exposure event. This will be divided into two parts: creating a semantic description of an exposure event, and linking that event to an adverse outcome. This use case was inspired by the AOP [45,46], which describes an exposure event, and the subsequent “key events”, which detail what happens from the exposure to the adverse outcome. The AOP framework is important because it aligns mechanistic steps with a relevant adverse biological outcome. Developing semantics around AOPs will enable the use of model organism data to generate hypotheses about missing links in AOP chains and make inferences across the data. Semantic representation of AOPs can provide a more holistic vision of exposures and their outcomes.

#### 3.7.2. Workshop Discussion

The goal of the use-case session was to update the proposed semantic model for an exposure event to explicitly include the measurements used in AOPs, specifically, biomarkers and their relevant metadata and contextual data. Participants agreed that exposure events and molecular initiating events (MIE) are two different concepts, with the exposure event preceding the MIE. Cigarette smoking is an example that could be used to examine this. Biomarkers can be very complex and include markers of susceptibility (such as epigenetic markers), physical and chemical properties of stimuli, and signatures that indicate routes and sources. Quantitative measurements of the biomarker and its timing, dose metrics, the magnitude and frequency of exposure, co-exposures, age and the stage of development, sex, and chemical interactions were all identified as important for describing an exposure event and AOP. Participants noted the potential utility of omics signatures and the importance of separating the marker from what the marker represents.

#### 3.7.3. Results: Future Directions for This Use Case

The group identified four potential scientific use cases to explore, all centered around the adverse outcome of asthma, which results from exposure to (1) particulate matter 2.5 (PM_2.5_), (2) carbon monoxide, (3) smoking, or (4) phthalates. Each scientific use case should include AOP, omics data, a large cohort, and a model system database.

Participants identified several important gaps:

- Absence of methods to annotate/link laboratory data with ontologies.
- Uncertainty around what numerical or statistical models are needed to analyze the data.
- Unknown or unidentified biomarkers.
- Difficulty in developing examples that include all important aspects of biomarkers.
- Uncertainty around how to disseminate complex results, especially concerning omics data.

Challenges identified by the group were mostly data-related, revolving around collecting, accessing, and integrating the necessary heterogeneous data. Defining the scope of the use case will be essential to reducing the issues of scale and dimensionality to a tractable level.

### 3.8. Additional MURAL Brainstorming: Gaps and Areas for Research

In addition to the four use-case discussions, participants also had the opportunity during a breakout MURAL session (Supplementary Materials S1) to provide input on additional semantic needs, gaps, and strategies that the Collaborative could work on, as shown in Table 3.

## 4. Theme 2: Building a Sustainable Community

### 4.1. Goals and Objectives of the Theme

Like for the use cases, a small WG convened in early 2020 to begin the process of crafting draft governance elements as a starting point for discussion at the September workshop. Development of a sustainable leadership and governance structure was imperative to allow the necessary ongoing collaborative activities to maintain progress. Building on that work, the goals of the workshop were to:

- Explore community interest in contributing to a collaborative effort.
- Determine the model for forming and sustaining a collaborative effort.
- Building on the goals, the objectives of the workshop were to:
- Develop an endorsed mission and vision statements for the Collaborative.
- Discuss challenges for the Collaborative as well as ideas for defining and ensuring success for the Collaborative.
- Present and discuss a proposed community model for the Collaborative, consider alternative models, and reach an endorsed model with which to move forward.
- Discuss and develop ideas for how the community model will work in practice and be sustained in the long term.

### 4.2. Strategic Elements—Vision, Mission, Goals, and Roles

**Figure 5:**
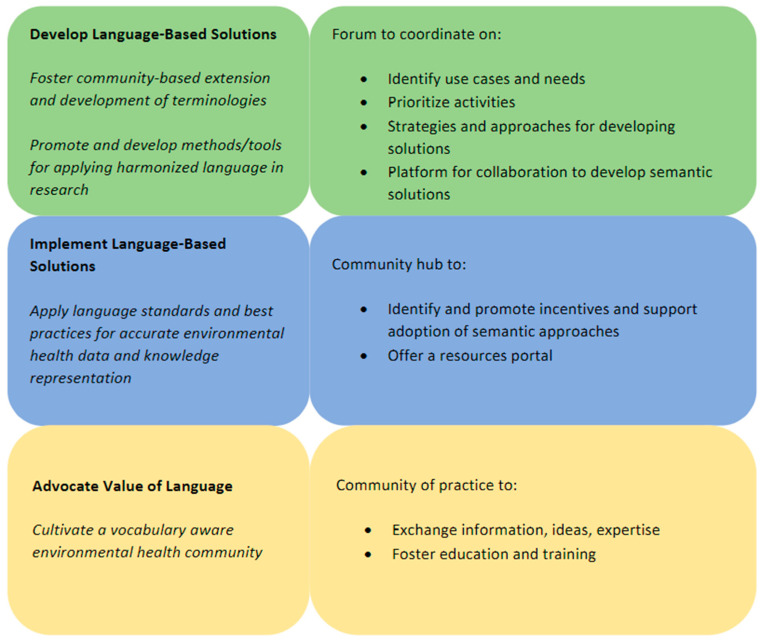
Proposed goals and roles of EHLC.

The first community building session introduced the draft vision and mission statements as well as strategic goals and the role that EHLC could play. An organization’s vision focuses on what it aspires to achieve in the long run or what will have changed if the organization is successful. The Collaborative’s proposed vision is to *leverage community-driven environmental health language standards to catalyze knowledge-driven discovery and improve public health*. Supporting the vision is the mission or fundamental purpose of the organization. EHLC’s proposed mission is to *advance integrative EHS research by developing and promoting adoption of a harmonized language*. To pursue that mission, the Collaborative proposed three high-level strategic goals, along with roles that EHLC could play in supporting those goals (Figure 5).

For each strategic element, a Zoom poll was used to gauge the level of participant endorsement of the above. Using a scale from 1 (low) to 5 (high), an element was considered endorsed if the total votes of 4–5 were greater than those of 1–3. While participants were highly supportive overall, the mission and strategic goals had less support and prompted more discussion (Table 4).

After voting, the attendees were asked to contribute their input on each of the strategic elements to the Community Building MURAL Board (Supplementary Materials S2). The input included wanting clarification of terms being used in the statements, wordsmithing of existing terms, suggesting new concepts to add, and even suggesting completely rewritten vision and mission statements. Participants were asked to indicate their support for a comment by placing stars next to the comments that resonated with them.

A few key themes emerged from the comments contributed. From an organizational perspective, EHLC would benefit from developing core values as well as long-term goals to guide the operations of the Collaborative. In addition, it needs to facilitate communication to interdisciplinary audiences, to ensure all stakeholders participate in consensus building. Furthermore, its activities should focus on data harmonization, integration, and interoperability, thereby helping to increase the impact of research by moving beyond knowledge discovery to interpretation and understanding. EHLC also needs to create more partnerships to incorporate EHS data into existing systems, as well as ensure its activities are synergistic with similar efforts. Finally, EHLC plays a role in offering education and training to help increase the adoption of semantic approaches.

### 4.3. Community Model

The second community-building session presented the draft community model for how the Collaborative could be organized. One key component of the model is a possible role for the Research Data Alliance (RDA) [69], to provide infrastructure for the Collaborative’s activities.

**Figure 6:**
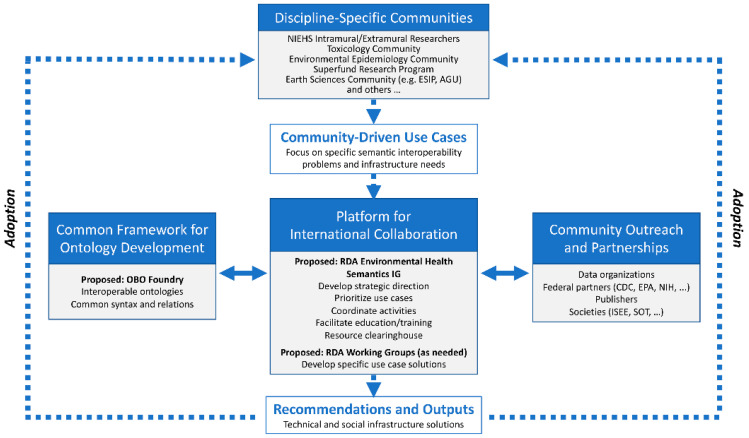
Draft community model for the organization of the Collaborative.

The proposed model (Figure 6) begins with individuals and/or groups from discipline-specific communities generating use cases based on research questions of interest. These use cases represent the need for harmonized language solutions that will enhance the findability, sharing, and interoperability of EHS data.

The use cases will be brought to a proposed RDA Environmental Health Semantics Interest Group (IG). This IG will provide a platform for the overall coordination of and collaboration between interested members. Its goal will be to design a strategic direction for developing and adopting language solutions, identify and prioritize use cases, coordinate activities, and form a Community of Practice space for exchanging information, offering a resource portal, and fostering education and training.

An RDA WG could be formed whenever a work product needs to be developed. If the product is an ontology, then ideally its development would follow the OBO Foundry framework to make it interoperable with other ontologies.

The IG and WG will work in concert with other relevant communities or partner organizations toward the development and implementation of any recommendations and outputs. Those products will be communicated back to the discipline-specific communities with the anticipation of their adoption.

As in the first community-building session, time was provided to answer clarifying questions and to post comments on the Community Building MURAL Board (Supplementary Materials S2). Key takeaways from the discussion and MURAL comments include:

- If RDA is to play a role, there is a need for significant awareness-raising within the EHS community about RDA. A Zoom poll of the workshop attendees indicated that 23% had not heard of RDA and 40% had heard of RDA but were not familiar with what it does.
- There is concern regarding potential need to pay for RDA membership.
- There is a need to ensure the community structure engages diverse practitioners, stakeholders, end users, etc.

### 4.4. Building and Sustaining the Community

The final community session offered an opportunity for participants to provide MURAL input (Supplementary Materials S1) on the four questions shown in Table 5. During this activity, the participants identified several activities to start in the near term, which aligned with strategic discussions and use-case sessions. Of primary importance is making progress on existing use cases and achieving a deliverable, to show success and inspire participant investment. Achieving that success will involve having a clear understanding of the use-case need, leveraging existing resources where possible, and developing solutions that can be readily incorporated into a researcher’s workflow. Another important activity is to implement a comprehensive outreach plan to not only expand representation and participation within EHLC but also ensure that relevant stakeholder groups are kept informed and potential partnerships/collaborations are developed. Finally, community members support offering more education and training to help researchers in the field become more “vocabulary aware”.

**Figure 7:**
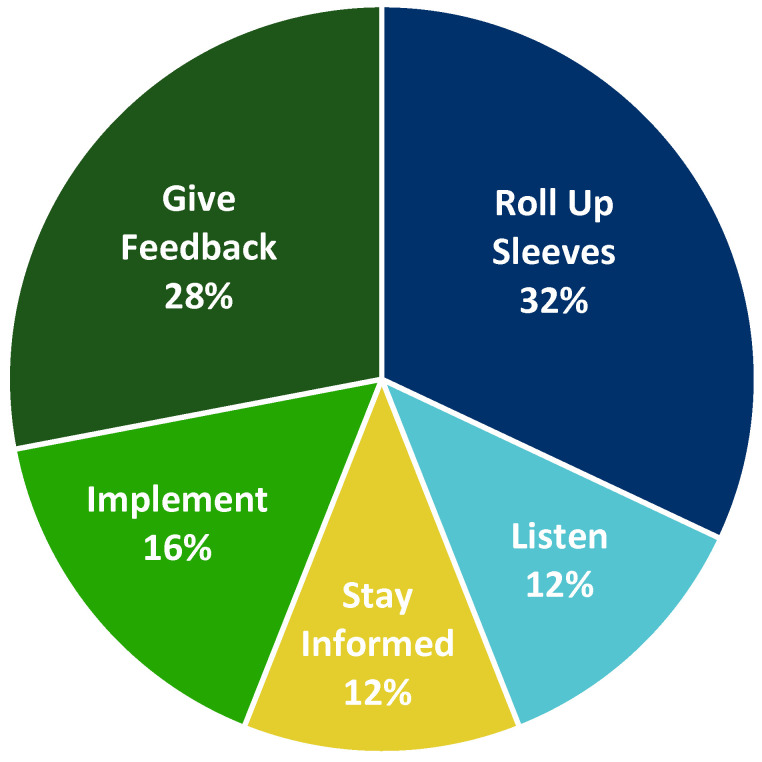
Levels of participant availability for ongoing collaboration.

Another poll at the close of the workshop asked participants about the level of involvement they would be able to provide. Levels of involvement that participants could select included “Listen” (listen in on future collaborative sessions), “Stay Informed” (keep abreast of developments in the collaborative effort), “Give Feedback” (offer comments, concerns, and opinions to shape the discussion), “Implement” (put harmonized language solutions into practice), and “Roll Up Sleeves” (commit to high-level active collaboration in language harmonization). The results of the poll are summarized in Figure 7 below.

While polling on the strategic elements (vision, mission, goals, and role) of EHLC reached the predefined levels for endorsement, the level of interest in these topics and the quality of suggestions suggested that additional refinement could be beneficial to the community’s success. As such, a community-building WG was subsequently formed to refine the elements based on workshop feedback and to conduct a future second round of community input and endorsement.

## 5. Results and Discussion

The workshop set out with the primary objective to begin the process of obtaining community agreement on the purpose, structure, and activities of the Collaborative. Based on the discussions, comments, and votes conveyed during the workshop, members of the EHS community have shown strong support for EHLC and provided the following key takeaways with respect to the workshop’s two goals.

**Developing Sustainable Language Solutions**—Attendees understand and confirm the value of applying harmonized language solutions in EHS research. A use-case approach is central to developing useful solutions because use cases represent real-world needs. The first phase of efforts should focus on identifying what resources currently exist and whether they can be used or extended to meet the need. Any development of a semantic solution needs to ensure the representation of diverse subject matter expertise and skillsets. There is also a need to develop methods and tools that can more easily incorporate semantic approaches into researcher workflows, to reduce the level of effort.

The level of participation in the pre-workshop and workshop use-case sessions is an endorsement of the relevance of these use cases. Use-case champions and members continue to work on these use cases, and the following best practices/guidelines have emerged to strengthen the work of the use cases.

- Identify a “Use-Case Champion”, a self-identified expert volunteer, to lead the group as a unique way to encourage both community participation and buy-in.
- Coordinate initiatives across use cases to prevent duplication and promote synergy.
- Define a clear scope to ensure outcomes are well-defined and reasonable.
- Work with large studies and datasets that can help increase adoption.
- Understand the needs and perspectives of the stakeholders, e.g., analysts, modelers, data generators, and developers.
- Reach out to the community to capture translational applications.
- Ensure transdisciplinary representation and relevant subject matter expertise are reflected in the use cases.
- Build on existing frameworks (e.g., AOPs, OBO) and ensure positive alignment with other standards-related efforts.
- Understand where artificial intelligence and machine learning (AI/ML) approaches may help in harmonizing different languages.

Despite these guidelines, subsequent work to develop effective models for executing use cases is still needed, e.g., to define use cases at the appropriate level of granularity, to form effective WGs, to define useful outcomes and products, and to develop engagement with related efforts.

Building solutions is only half of the success equation; the other half is ensuring the adoption/implementation of the solutions. Attendees showed a clear interest in EHLC activities to address ways to overcome the social and technical barriers to adoption. Education and training were considered important to raise awareness about the value and need for a harmonized language, about semantic approaches broadly (e.g., developing, selecting, and using ontologies), and about how harmonized language may help fulfill the objectives of the NIH 2023 data-management and -sharing plans. Targeted education and training opportunities were frequently mentioned, especially around awareness and the use of ontologies as well as tools for generating and using metadata.

**Building a Sustainable Community**—At the core of any community-driven effort is the need for volunteers to participate and keep the momentum going. Most workshop attendees expressed an interest in EHLC and indicated various levels of willingness to participate. EHLC needs to engage in broader outreach efforts to expand its participant base and ensure it represents the multidisciplinary nature of EHS research. In addition, formal and informal communications with other relevant stakeholders and partners need to be developed. To keep members engaged, EHLC needs to create an open, collaborative space and, most importantly, show it is having an impact.

The participants also endorsed the need for a community-developed, interactive resource portal. The portal would be a platform for centralizing resources such as training offerings, recorded webinars, relevant EHS language standards, and ontology development tools, among other resources useful to the EHS community in developing and implementing harmonized language approaches. Future Collaborative meetings will begin addressing the next steps in these two areas.

As part of the Collaborative’s goal to raise awareness of existing resources, EHLC hosted a webinar in December 2021 entitled, “*Knowledge Bases as a Tool to Understand the Intersection of Genes, Phenotypes, and Environment*”, led by Julie McMurry, University of Colorado Anschutz (Recording Available) [71]. Use-case groups are also engaging the large number of workshop participants who were interested in ongoing participation (Figure 7). The “Results: Future Directions” section of each of the use cases identified low-hanging fruits, and these are currently being explored to keep the momentum going.

## 6. Conclusions

There is a collective recognition that the lack of a harmonized language to describe environmental health data, findings, and knowledge is impeding our ability to advance research and to make informed policy decisions. Although numerous efforts either exist or are under development related to minimal information standards, ontologies, etc., no initiative exists specifically addressing the needs of the environmental health community. EHLC was created to provide a continuous forum for community members to identify needs, coordinate action, and either extend existing or develop new solutions. This forum offers a space for subject matter experts, data curators, ontologists, systems developers, policymakers, and other stakeholders from the environmental health and toxicology fields as well as related disciplines to participate and collaborate and allows for the self-selection of individuals to spearhead these efforts. Diverse discipline and expertise representation is needed to ensure that solutions are developed to meet community needs and enable maximum community adoption. Hosting the workshop was key to the launch of EHLC as it provided an opportunity to garner input from a wide range of stakeholders with different backgrounds, gain insight into important EHS challenges that need to be addressed, obtain answers to specific questions, identify best practices to support future EHLC initiatives, and allow for the self-identification of community members interested in continuing these efforts moving forward.

In summary, the outcomes of the workshop refined the mission of EHLC and advanced efforts to improve public health by providing a continuous and collaborative forum to:

- Move the field toward community-endorsed best practices.
- Break down current silos, support a common infrastructure, and interconnect data-resource ecosystems.
- Leverage existing ontologies (HHEAR, ENVO, ECTO, EXO, etc.) as well as advance new semantic approaches when needed.
- Promote best practices in data management and sharing, such as FAIR principles.
- Catalyze knowledge-driven discovery by facilitating AI/ML approaches—fully AI ready.

## Figures and Tables

**Table 1:** Developing sustainable language solutions: Use cases.

Use Case	Use Case Title	Use Case Champion
Discovery of exposure data	What data exist for a given chemical/endpoint/exposure scenario?	Michelle Angrish, US EPA
Place-based exposures	Data and tools needed to harmonize place-based health research	Carmen Marsit, Emory University
Integration of exposure data	Combine individual-level data from multiple independent studies to understand how exposures X + Y impact health outcome Z	Jeanette Stingone, Columbia University
Bridging exposure and biomarkers of exposure	What are the biological processes and biomarkers associated with exposure and how do they relate to the potential for an adverse outcome associated with a given exposure?	Stephen Edwards, RTI and Chirag Patel, Harvard University

**Table 2:** Key “actors” or stakeholders identified during workshop.

**Data generators**: individuals executing the studies and reporting the primary findings	Less familiar with computational approaches aimed at reusing and connecting data High level of subject matter expertise May perceive low value in making data accessible and interoperable
2. **Data consumers:** those who are searching for information	Wide range of ability to find and connect data May have limited expertise on terminology or methodology Highly values data interoperability and accessibility
3. **Data modelers**: those who are seeking to create new information based on existing data	More familiar with approaches for reusing and connecting data Lower expertise on terminology or methodology Highly values data interoperability and accessibility
4. **Resource developers**: those who are developing tools and databases that aid in finding or interacting with data	High familiarity with approaches to connecting data Wide range of subject matter expertise Highly values making data within resources accessible and interoperable

**Table 3:** Attendee feedback on three questions about semantic needs, gaps, and strategies for future research efforts.

What gaps/pain points/challenges would you like to propose be worked on in the Collaborative?	Establish a formal WG of international regulatory agencies and regulated stakeholders to develop extensions to the Organization for Economic Cooperation and Development (OECD) [47] Harmonized Templates (OHTs) for formal submission to the OECD for review and acceptance. Consider ways to harmonize environmental health data with other NIH institutes and centers. Develop a taxonomy in exposure science and a harmonizing structure in each subdomain. Identify what is missing from electronic health records that is needed for EHS research. Understand how standards are embodied in generalist versus specialized repositories and what is needed to facilitate the discovery of EHS research. For existing ontology/vocabulary terms, inventory how different community domains understand a term and the context in which they apply it.
What data/terminology standards and/or tools are you currently using for data query and aggregation?	Platforms—CTD [48], Health Assessment Workspace Collaborative (HAWC) [49], Integrated Chemical Environment (ICE) [50], Toxic Exposome Database [34], ToxRefDB [51]. Programming/query languages—Python [52], SPARQL [53], SQL [54]. Standards—OHTs, Cross-Domain Observational Metadata Environmental Sensing Network (X-DOMES) [55]. Terminologies and ontologies—AOP Ontology [56], BioAssay Ontology (BAO) [57], Environment Ontology (ENVO) [58], Exposure Ontology (EXO) [59], Medical Subject Headings (MeSH) [60], Ontology for Biomedical Investigations (OBI) [2], Unified Code for Units of Measure (UCUM) [61], Unified Medical Language System (UMLS) [62]. Terminology portals—Bioportal [63], OBO Foundry [38], Ontobee Browser [64]. Tools—Chemlistem [65], EQUIS [66], MedCAT [67], PubTator [68].
Where do terminologies need to be harmonized? What terminology gaps exist? Which terminologies should be endorsed for EHS-related use?	Gaps and areas of harmonization include incorporating social determinants of health into EHS research, agreement on and adoption of chemical substance identifiers, fuller representation of ecotoxicology, and the need to coordinate with international bodies (OECD, WHO, etc.). Endorsement suggestions were few but included: AOP ontology, OHTs (recognizing their gaps), Clinical Data Interchange Standards Consortium (CDISC), and NIH Common Data Elements (CDEs).

**Table 4:** Community endorsement of EHLC strategic elements.

	1 (Low)	2	3	4	5 (High)
Vision	-	8%	24%	51%	17%
Mission	2%	17%	29%	40%	12%
Strategic goals	5%	8%	26%	53%	9%
EHLC roles	-	4%	25%	61%	11%

**Table 5:** Attendee feedback on four questions about future EHLC efforts.

**What would you like to see the community work on/accomplish in the next 6–12 months?**	Continue the community-building process—Convene a small group to develop an outreach plan to expand the community. Identify what needs to exist—Based on use cases, identify language needs; conduct a scoping review to identify the gaps and determine the missing resources and tools. Leverage what exists—Inventory what exists, identify what is relevant for EHS needs, and compile resources in a community-maintained interactive portal. In addition, liaise with parallel activities to incorporate EHS needs and avoid duplicating efforts. Develop methods and tools—Build “plug and play” tools that easily fit into the researcher workflow; create data pipelines between existing data silos. Focus on education and training—Conduct a community knowledge assessment of key ontologies and standard vocabularies as well as other training needs; establish coaching opportunities to facilitate cross-communication between domain and technical experts.
**How can the Collaborative support the creation of a more “vocabulary-aware” EHS research community?**	Create a Community of Practice space—Offer “birds of a feather” small informal group discussions to share both successful and unsuccessful projects as educational examples. Launch outreach efforts—Have EHLC members help spread the word, engage multiple channels (societies, publishers, government agencies), and use social media to keep relevant groups informed. Support training—Offer a “fellowship” program of coaching opportunities to connect subject matter experts with technical specialists, host short but frequent brown bag hands-on sessions, and produce short educational videos.
**What are the barriers to adoption? What can the Collaborative do to promote the adoption of harmonized language approaches?**	Technical barriers—Use of harmonized language approaches is often too time intensive due to the lack of resources and tools to incorporate semantic solutions into the workflow. Social barriers—There is a lack of understanding of what standard terminologies and ontologies are and the value of using them. In addition, the benefit of using them is very low for an individual researcher compared to someone performing large data integration or developing data systems. EHLC response—EHLC activities can address the barriers and promote adoption by supporting resources and training to inform people where to start, providing concrete examples of how semantic standards have facilitated research, offering coaching opportunities akin to the OECD AOP Development Programme [70], and creating a space to facilitate cross-disciplinary understanding.
**How do we define success for the Collaborative? How can we measure it?**	Outcome-based—EHLC will be successful when there is less need for harmonization because applying semantic solutions will become the norm and readily incorporated into the researcher workflow. EHLC participation metrics—Number of members involved in the Collaborative, retention, organizational support for participation, and participation levels in training. Other metrics—Adoption of semantic approaches in EHS research as tracked via the citation of ontologies and use of templates.

## Data Availability

Not applicable.

## Supplementary Materials

The following supporting information can be downloaded at: https://www.mdpi.com/article/10.3390/ijerph20032317/s1, Supplementary Materials S1: EHLC Workshop—Community Breakout Sessions; Supplementary Materials S2: ELC Workshop—Community Building.
