# Supplementary material for: Workshop Report: Catalyzing Knowledge-Driven Discovery in Environmental Health Sciences through a Harmonized Language
Source: Int J Environ Res Public Health. 2023 Jan 28;20(3):2317. doi: 10.3390/ijerph20032317 (PMC9915042; doi:10.3390/ijerph20032317)
Supplement: Supplementary file 1 [file Holmgren et al_EHLC Workshop_Supp S1-Community Breakout Sessions.pdf › Holmgren et al_EHLC Workshop_Supp S1-Community Breakout Sessions.pdf]

EHLC
The Environmental Health Language Collaborative

Community Input - Semantic Solutions

Thursday, September 9
2:45 - 4:15 pm EDT

MURAL INSTRUCTIONS

LOGISTICS & TIPS

As with all tools, there's a learning curve! Be patient with yourself, others, and technology. Here are some helpful tips to navigate Mural with confidence.

Tech stuff

A Chrome browser is preferred (but it also works in MS Edge). Sometimes you'll need to refresh!

Avatar

You may have entered the board as an animal, with initials, or with an image. This is normal! We like to keep our feedback anonymous.

New User Tip

Double-click in the middle of a sticky note to activate the typing function. When you're done, click on any white space around your sticky to close it out.

The Basics

Use your trackpad, mouse, or the Zoom toolbar in the bottom right of your screen to move. To view other areas of the workspace, you can left click any white space on the board, continue holding, and drag to your view of choice.

Get help

E-mail Canden Byrd at NTP-Meetings@icf.com if you have any issues!

1 What gaps/pain points/challenges would you like to propose be worked on in the Collaborative?

Use the sticky notes below to add your answer to the question. Double click in the sticky note to add content. When you're done, click on any white space around your sticky to close it out.

Drag a star icon to important feedback you'd like to emphasize.

2 For you to become engaged, what would you like to see the community work on/ accomplish in the next 6-12 months?

Use the sticky notes below to add your answer to the question. Double click in the sticky note to add content. When you're done, click on any white space around your sticky to close it out.

Drag a star icon to important feedback you'd like to emphasize.

3 What data/terminology standards and/or tools do you most commonly use (or have used) for data query and aggregation? What standards or tools are missing that are needed?

Use the sticky notes below to add your answer to the question. Double click in the sticky note to add content. When you're done, click on any white space around your sticky to close it out.

Drag a star icon to important feedback you'd like to emphasize.

4 Where do terminologies need to be harmonized? What specific terminology gaps exist? Which terminologies should be endorsed for EHS-related use?

Use the sticky notes below to add your answer to the question. Double click in the sticky note to add content. When you're done, click on any white space around your sticky to close it out.

Drag a star icon to important feedback you'd like to emphasize.

EHLC
The Environmental Health Language Collaborative

Community Input - Collaborative Community

Friday, September 10
12:00 - 1:30 pm EDT

MURAL INSTRUCTIONS

LOGISTICS & TIPS

As with all tools, there's a learning curve! Be patient with yourself, others, and technology. Here are some helpful tips to navigate Mural with confidence.

Tech stuff

A Chrome browser is preferred (but it also works in MS Edge). Sometimes you'll need to refresh!

Avatar

You may have entered the board as an animal, with initials, or with an image. This is normal! We like to keep our feedback anonymous.

New User Tip

Double-click in the middle of a sticky note to activate the typing function. When you're done, click on any white space around your sticky to close it out.

The Basics

Use your trackpad, mouse, or the Zoom toolbar in the bottom right of your screen to move. To view other areas of the workspace, you can left click any white space on the board, continue holding, and drag to your view of choice.

Get help

E-mail Canden Byrd at NTP-Meetings@icf.com if you have any issues!

1 How do we want to define success for the Collaborative? How can we measure it?

Use the sticky notes below to add your answer to the question. Double click in the sticky note to add content. When you're done, click on any white space around your sticky to close it out.

Drag a star icon to important feedback you'd like to emphasize.

2 What would you like to see the community work on/accomplish in the next 6-12 months?

Use the sticky notes below to add your answer to the question. Double click in the sticky note to add content. When you're done, click on any white space around your sticky to close it out.

Drag a star icon to important feedback you'd like to emphasize.

3 What are the barriers to adoption? What can the Collaborative do to promote adoption of harmonized language approaches?

Use the sticky notes below to add your answer to the question. Double click in the sticky note to add content. When you're done, click on any white space around your sticky to close it out.

Drag a star icon to important feedback you'd like to emphasize.

4 How can the Collaborative support creating a more "vocabulary aware" EHS research community?

Use the sticky notes below to add your answer to the question. Double click in the sticky note to add content. When you're done, click on any white space around your sticky to close it out.

Drag a star icon to important feedback you'd like to emphasize.
