# Supplementary material for: Workshop Report: Catalyzing Knowledge-Driven Discovery in Environmental Health Sciences through a Harmonized Language
Source: Int J Environ Res Public Health. 2023 Jan 28;20(3):2317. doi: 10.3390/ijerph20032317 (PMC9915042; doi:10.3390/ijerph20032317)
Supplement: Supplementary file 1 [file Holmgren et al_EHLC Workshop_Supp S2-Community Building.pdf › Holmgren et al_EHLC Workshop_Supp S2-Community Building.pdf]

EHLC
The Environmental Health Language Collaborative

Community Development

To kickstart the conversation on community development at the workshop, the following community vision, mission, goals, roles, and model are being proposed for community input.

1 MURAL INSTRUCTIONS

LOGISTICS & TIPS

As with all tools, there's a learning curve! Be patient with yourself, others, and technology. Here are some helpful tips to navigate Mural with confidence.

Tech stuff

A Chrome browser is preferred (but it also works in MS Edge). Sometimes you'll need to refresh!

Avatar

You may have entered the board as an animal, with initials, or with an image. This is normal! We like to keep our feedback anonymous.

New User Tip

Double-click in the middle of a sticky note to activate the typing function. When you're done, click on any white space around your sticky to close it out.

The Basics

Use your trackpad, mouse, or the Zoom toolbar in the bottom right of your screen to move. To view other areas of the workspace, you can left click any white space on the board, continue holding, and drag to your view of choice.

Get help

E-mail Canden Byrd at NTP-Meetings@icf.com if you have any issues!

INSTRUCTIONS

- Review the Environmental Health Language Collaborative's community vision, mission, goals, roles, and governance in Sections 2-6.
- Provide your feedback in the provided sticky notes for each section. Feel free to add as much feedback as you'd like.
- If you see feedback provided that you'd like to re-emphasize, drag the star icons below the sticky notes to the sticky note you'd like to emphasize.

LET'S BREAK THE ICE - WHAT IS YOUR FAVORITE FOOD?

New user tip
Double-click in the middle of a sticky note to activate the typing function. When you're done, click on any white space around your sticky to close it out.

Categorize your answer!

Hot Food | Cold Food

Drag a star icon to the food you also enjoy!

2 COMMUNITY VISION

What are we aspiring to achieve?

What changes do you recommend be made to the vision that would lead to your endorsement?
How might we improve the community vision? What would you change and why?

Use the sticky notes surrounding the vision to provide feedback. Double click in the sticky note to add content.

The **vision** of the Environmental Health Language Collaborative is to leverage community-driven environmental health language standards to catalyze knowledge-driven discovery and improve public health.

There's no room! I need to add more sticky notes. Double click beside another sticky note to add a new one of the same size and color!

Drag a star icon to important feedback you'd like to emphasize.

3 COMMUNITY MISSION

Why do we exist?

What changes do you recommend be made to the mission that would lead to your endorsement?
How might we improve the community mission? What would you change and why?

Use the sticky notes surrounding the mission to provide feedback. Double click in the sticky note to add content.

The Collaborative's **mission** is to advance integrative environmental health research by developing and promoting adoption of a harmonized language.

There's no room! I need to add more sticky notes. Double click beside another sticky note to add a new one of the same size and color!

Drag a star icon to important feedback you'd like to emphasize.

4 COMMUNITY GOALS

How will we achieve our mission/vision? What do we have to do?

A. What changes do you recommend be made to the goals that would lead to your endorsement?
How might we improve the community goals? What would you change and why?

Use the sticky notes surrounding each goal to provide feedback. Double click in the sticky note to add content.

1. Develop Language-Based Solutions
Foster community-based extension and development of knowledge organization systems

Promote and develop methods/tools for applying harmonized language in research

2. Implement Language-Based Solutions
Apply language standards and best practices for accurate environmental health data and knowledge representation

3. Advocate Value of Language
Cultivate a vocabulary-aware environmental health community

There's no room! I need to add more sticky notes. Double click beside another sticky note to add a new one of the same size and color!

B. What additional goals should we consider including and why?

Drag a star icon to important feedback you'd like to emphasize.

5 COMMUNITY ROLES

A. What changes do you recommend be made to the roles/activities that would lead to your endorsement?
How might we improve the community roles? What would you change and why?

Use the sticky notes surrounding each role to provide feedback. Double click in the sticky note to add content.

1. Forum to collaborate on: (1) Identifying use cases and needs, (2) Prioritizing activities, and (3) Developing strategies and approaches for developing solutions

Serve as platform for collaboration to develop semantic solutions to address identified needs

2. Centralized community core to:
Identify and promote incentives and support adoption and use of semantic approaches

Identify and apply metrics to gauge success

Offer a resource clearinghouse

3. Community of practice to: (1) Exchange information, ideas, and expertise, and (2) Foster education and training

There's no room! I need to add more sticky notes. Double click beside another sticky note to add a new one of the same size and color!

B. What additional roles/activities should we consider including and why?

Drag a star icon to important feedback you'd like to emphasize.

5a COMMUNITY VALUES

What do we stand for?

What guiding values/principles/beliefs would you like the Collaborative to uphold and represent?

Use the sticky notes surrounding each role to provide feedback. Double click in the sticky note to add content.

There's no room! I need to add more sticky notes. Double click beside another sticky note to add a new one of the same size and color!

Drag a star icon to important feedback you'd like to emphasize.

6 COMMUNITY MODEL

A. How might we improve the community model? What would you change in the model and why?

Double click the image below to learn more about the proposed community model from the community website.
Use the sticky notes surrounding the model to provide feedback. Double click in the sticky note to add content.

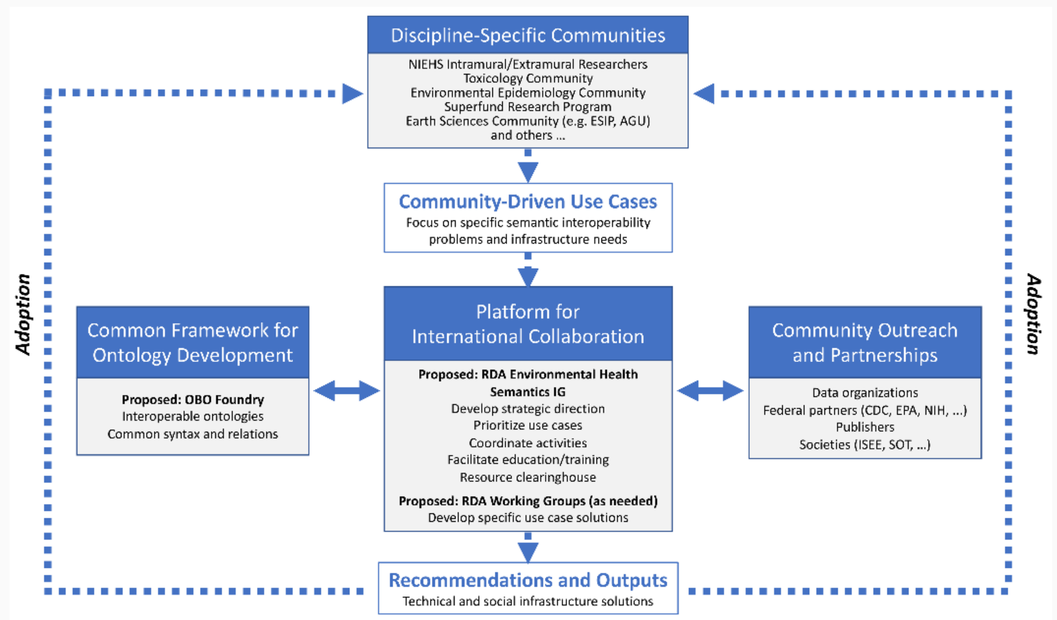

There's no room! I need to add more sticky notes. Double click beside another sticky note to add a new one of the same size and color!

Drag a star icon to important feedback you'd like to emphasize.

B. What additional governance issues should we consider including and why?

C. What governance issues/activities should be worked on/achieved in the next 6-12 months?

D. What other successful communities have you participated in that would provide an alternative model to RDA?

E. What would be the best technical platform(s) to enable creating community and doing collaborative work? e.g., listserv, wiki, Teams, other?
